# Green synthesis of copper nanoparticles: a promising solution for drug resistance and cancer therapy challenges

**DOI:** 10.1186/s43046-024-00254-y

**Published:** 2024-12-28

**Authors:** Anand G. Krishna, S. Sahana, H. Venkatesan, Vettrivel Arul

**Affiliations:** 1 Department of Organon of medicine, Vinayaka Mission’s Homoeopathic Medical College and Hospital, Vinayaka Mission’s Research Foundation (DU), Salem, India; 2 Department of Practice of Medicine, Vinayaka Mission’s Homoeopathic Medical College and Hospital, Vinayaka Mission’s Research Foundation (DU), Salem, India; 3 Department of Community Medicine, Vinayaka Mission’s Homoeopathic Medical College and Hospital, Vinayaka Mission’s Research Foundation (DU), Salem, India

**Keywords:** Copper nanoparticles, Green synthesis, Biomedicine, Antibacterial activity, Anticancer potential, Drug delivery, Active delivery, Passive delivery, Cytotoxicity

## Abstract

Green synthesis techniques have drawn a lot of interest lately since they are beneficial to the environment and have potential uses in a variety of industries, including biomedicine. Because of their special physicochemical characteristics, copper nanoparticles (CuNPs) have become one of the most interesting options for use in biological applications among nanomaterials. An overview of green synthesis methods for CuNPs is given in this review, along with a discussion of their applications in cancer therapeutics. The benefits and drawbacks of certain green synthesis techniques, such as plant-mediated, microorganism-mediated, and other environmentally friendly processes, are discussed. Moreover, a thorough discussion is given of CuNPs’ biological uses, including their antibacterial activity, anticancer potential, drug transport, and bioimaging capabilities. Furthermore, difficulties and prospects for the application of green-synthesised CuNPs in biomedicine are discussed.

## Introduction

In 2040, there are expected to be 28.4 million cancer cases across the globe, a 47% increase from 2020. A widespread lack of growth control accompanies the continuous progression of a complicated group of symptoms known as cancer. Globally, GLOBOCAN estimates that there were 19.3 million births about 10 million cancer-related deaths and cases globally. Global cancer incidences are expected to reach 28.4 million in 2040, up 47% from 2024. Using a projected 2.3 million new instances of breast cancer in women, it has surpassed lung cancer being the most common type of cancer diagnosed, then by stomach, colon, lung, and prostate cancers, and reported 1.8 million fatalities, with lung cancer continuing to be the most common cause of death from cancer, followed by liver, colorectal, stomach, as well as breast cancer in women. As this illness is tissue-based, diagnosing it and effectiveness of treatment is a serious limitation due to tissue variance [1].

By providing new instruments and materials for cancer monitoring, diagnosis, and treatment, nanotechnology has transformed several industries, including medical. Since they exhibit exceptional conductivity, specificity, sensitivity, a high surface area-to-volume ratio, and catalytic activity, copper nanoparticles (CuNPs) have attracted a lot of interest among nanomaterials [2]. CuNPs are therefore attractive candidates for a range of biological uses, such as drug delivery systems, bioimaging agents, antibacterial agents, and anticancer treatments [3].

Green synthesis techniques have surfaced as viable substitutes for conventional chemical synthesis methods in the production of nanoparticles. Using natural sources, such bacteria, plants, and other eco-friendly materials, to reduce and stabilise metal ions into nanoparticles is known as ‘green synthesis’ [4]. Green synthesis has a number of benefits over traditional techniques, such as being more affordable, scalable, and environmentally friendly. Furthermore, green-synthesised nanoparticles are appropriate for biomedical applications due to their improved biocompatibility and decreased toxicity [5].

We outline green synthesis methods for CuNPs in this review and briefly address their applications in the biomedical domain especially in cancer. We present a variety of green synthesis techniques, such as microorganism- and plant-mediated synthesis. We also go into great detail about the medicinal uses of CuNPs, including drug transport, bioimaging, antibacterial activity, and anticancer potential. Lastly, we discuss the difficulties and potential applications of green-synthesised CuNPs in biomedicine.

## Green synthesis of copper nanoparticles

In the process of creating green synthesis CuNPs, copper ions (Cu^2+^) are reduced utilising natural sources as stabilising and reducing agents, including microbial cultures, plant extracts, and other materials that are safe for the environment. Numerous green synthesis techniques have been established, and each has special benefits with regard to yield, control over size, and scalability [6].

### Synthesis mediated synthesis

Because they include phytochemicals such flavonoids, polyphenols, terpenoids, and alkaloid substances, which function as reducing and capping agents, plants are a great source for the production of nanoparticles [7]. Utilising aqueous extracts from various plant components, including leaves, stems, roots, and fruits, Cu^2+^ ions are reduced during the process of plant-mediated production of CuNPs. In order to create CuNPs, the plant extract is usually mixed with a copper salt solution at the right pH, temperature, and reaction time [8].

Many plant species, notably *Aloe vera*, *Azadirachta indica* (neem), *Ocimum sanctum* (holy basil), and *Camellia sinensis* (green tea), have been investigated for the green synthesis of CuNPs. These plants have a range of bioactive substances that help reduce copper ions and stabilise the resultant nanoparticles [4]. Polysaccharides and phenolic chemicals, for instance, are present in *Aloe vera* leaf extract and may efficiently convert Cu^2+^ ions to CuNPs and inhibit their agglomeration [9].

Plant-mediated synthesis has a number of benefits, including an easy synthesis procedure, readily available raw ingredients, and nanoparticles that are biocompatible. Moreover, the plant extracts may impart therapeutic qualities to the synthesised CuNPs, increasing their potential for use in biomedical applications [10].

### Microorganism-mediated synthesis

Microorganisms such as algae, fungus, and bacteria have also been used to produce CuNPs in an environmentally friendly manner. These microbes have metabolites and enzymes that effectively lower metal ions and speed up the creation of nanoparticles [10]. The process of producing CuNPs by microorganism-mediated synthesis usually entails soaking microbial cultures in a copper salt solution, which is then followed by the reduction of Cu2^+^ ions and the consequent creation of nanoparticles [11].

The synthesis of CuNPs by bacteria like *Escherichia coli*, *Bacillus subtilis*, and *Pseudomonas aeruginosa* has been the subject of much research. Reductases are among the many enzymes that these bacteria create, and they are essential for the reduction of metal ions [9]. Similar to this, because they secrete extracellular enzymes, fungus including *Aspergillus*, *Fusarium*, and *Penicillium* species have been used for the environmentally friendly production of CuNPs [6].

Benefits of microorganism-mediated synthesis include control over nanoparticle size and form, scalability, and speed of synthesis. Furthermore, the addition of biomolecules from microbial sources may result in improved stability and dispersibility for the resultant CuNPs [7]

### Other eco-friendly approaches

Various environmentally friendly methods have been investigated for the green synthesis of CuNPs, apart from the plant and microorganism-mediated synthesis. These include the use of bio-waste materials, including fruit peels and eggshell membranes, for the manufacture of nanoparticles and the reduction and stabilisation of natural polymers, like chitosan and starch [8].

As it may chelate metal ions and make their reduction easier, chitosan, a biocompatible and biodegradable polymer derived from chitin, has been used in the manufacture of CuNPs [4]. In a similar vein, given the right reaction circumstances, starch, a polysaccharide found in large quantities in plants, can act as a reducing agent for the production of CuNPs [9].

Fruit peels and eggshell membranes are examples of bio-waste materials that include bioactive chemicals that can efficiently decrease metal ions and stabilise the resultant nanoparticles. These materials are appealing for ecological nanotechnology applications because they provide the twin benefits of waste utilisation and nanoparticle production [10].

Overall, there are a number of environmentally acceptable methods for the green synthesis of CuNPs that have clear benefits in terms of biocompatibility, long-term viability and affordability. These techniques offer adaptable platforms for the production of CuNPs with specific characteristics needed for biomedical uses.

## Biomedical applications of copper nanoparticles

Because of their special physicochemical qualities, which include antibacterial capability, anticancer potential, drug transport capabilities, and bioimaging features, copper nanoparticles have a wide variety of medicinal uses.

In this part, we go over the various biological uses for CuNPs and emphasise how they may be used to solve important healthcare issues.

### Antibacterial activity

The public’s health is seriously threatened by the rise of bacteria that are resistant to antibiotics, which makes the creation of new antimicrobial agents necessary. Due to their strong antibacterial action against a wide range of pathogens, copper nanoparticles have become attractive options for treating bacterial infections [11]. CuNPs’ antibacterial action is mediated by the release of copper ions, which cause bacterial cells to undergo oxidative stress, membrane degradation, and protein denaturation, ultimately resulting in cell death [12].

CuNPs’ bactericidal effectiveness against clinically significant pathogens, such as *Escherichia coli*, *Pseudomonas aeruginosa*, *Staphylococcus aureus*, and methicillin-resistant *Staphylococcus aureus* (MRSA), has been shown in a number of investigations [9]. Furthermore, it has been demonstrated that CuNPs and antibiotics work synergistically to overcome bacterial strains’ resistance to antibiotics [6].

CuNPs’ antimicrobial properties have been investigated for use in disinfectants, implant coatings, and wound dressings, among other biomedical uses. CuNP-based wound dressings have the potential to expedite the healing process by inhibiting bacterial colonisation and decreasing inflammation [7]. Similarly, CuNP-containing implant coatings can enhance the long-term functionality of medical implants and reduce implant-associated infections [8]. To further lower the risk of nosocomial infections, CuNP-based disinfectants provide an efficient way to sterilise medical equipment and healthcare environments [4].

All things considered, CuNPs’ strong antibacterial activity makes them intriguing options for tackling the rising danger of antibiotic resistance and enhancing infection control in hospital environments.

### Anticancer potential

Since cancer is still one of the top causes of death globally, new treatment approaches must be developed. Because of their capacity to cause cytotoxicity in tumour cells while protecting healthy cells, copper nanoparticles have gained attention as possible therapies for cancer [9]. Copper (Cu) is a soft, pliable, readily bending metal with high thermal and electrical conductivities that is an essential part of metabolism in both plants and animals. In comparison to other transition metals like silver, gold, and platinum, copper nanoparticles (NPs) are more economically priced. Green synthesis of CuNPs has gained attention in recent years, and broccoli green extract has been hailed as an eco-friendly and green precursor for one-pot biosynthesis. Furthermore, a chitin-based silver and copper nanocomposite was discovered to have an inhibitory concentration (IC50) of 31 mg when tested for its cytotoxicity against HUMAN BREAST CANCER (MCF-7) cells. Additional research demonstrated a rise in ROS generation, a drop-in antioxidant enzyme activity, and a breakdown of membrane integrity, supporting the Cu–AgNPs-based nanocomposite’ cytotoxic effect on cancer cells [13].

CuNPs have anticancer properties through a variety of mechanisms, such as producing reactive oxygen species (ROS), interfering with cellular signalling pathways, and inducing apoptosis.[10].

CuNPs have been shown in preclinical trials to be effective against a variety of cancer types, including colon, prostate, lung, and breast cancer [11]. By taking advantage of cancer cells’ increased vulnerability to cellular oxidative stress and damage to DNA in comparison to normal cells, CuNPs selectively kill cancer cells [12]. Additionally, it has been demonstrated that the use of CuNPs in combination treatments can increase the effectiveness of traditional chemotherapy medications, hence overcoming drug resistance and increasing treatment results [9].

CuNPs have direct lethal effects, but they can also stop cancer from spreading by altering the processes of cell adhesion, migration, and invasion [6]. Moreover, CuNPs show promise for theragnostic uses, where they may be used to imaging as well as cancer treatment [7]. Specific targeting of cancer cells and real-time monitoring of therapy responses can be accomplished by conjugating targeting ligands or imaging agents to CuNPs [14]. Copper nanoparticles when combined with present drugs inhibit glutamine metabolism and enhance tumour cuproptosis, thereby it was sound facilitating the transformation of the tumour immunosuppressive microenvironment [8].

A study examines the use of CuNPs for the treatment of colon cancer. The authors found that copper nanoparticles had a strong cytotoxic effect on colon cancer cells in vitro and also had a potent inhibitory effect on the proliferation of colon cancer [15].

All things considered, CuNPs’ diverse anticancer potential offers hope for the creation of strong cancer treatments with increased efficacy and fewer adverse effects.

### Therapeutic drug delivery utility

Several studies have been carried out so far and several more are in progress to discover NP-based drug targeting design. These nanocarriers typically should possess certain fundamental characteristics such as (1) ability to remain stable in the vascular system (blood) until they reach their target, TME, (2) to escape the reticuloendothelial system (RES) clearance, (3) escape mononuclear phagocyte system (MPS), (4) accumulate in TME via tumour vasculature, (5) high-pressure penetration into the tumour fluid, and (6) reach the target and only interact with tumour cells [4]. One of the biggest obstacles in drug research is still getting therapeutic molecules to target tissues in an effective manner. Because of their large surface area-to-volume ratio, adjustable surface chemistry, and capacity to encapsulate a broad variety of pharmaceuticals, copper nanoparticles have become more attractive as drug delivery vehicles [16]. Both hydrophobic and hydrophilic medicinal products can be transported by CuNPs, which provide defence against degradation and regulated release at the intended site of action [9]. CuNP-based drug delivery systems have been designed using a variety of techniques, including as surface functionalization, encapsulation in polymeric matrices, and stimuli-responsive drug release processes [10]. CuNPs’ surface functionalization with targeting ligands, including peptides or antibodies, allows for targeted cell recognition and binding, which improves the effectiveness of drug administration [11]. Furthermore, spatiotemporal control over drug release is made possible by stimuli-responsive drug delivery systems, such as pH-responsive or light-responsive Products which reduce off-target effects and enhance therapeutic results [12].

CuNP-based systems for drug delivery have been studied for the delivery of antibiotics, nucleic acid-based therapies, and chemotherapeutic medications [9]. Enhanced bioavailability, extended circulation duration, and decreased systemic toxicity are only a few benefits of these systems [6]. Moreover, the blood–brain barrier and other biological barriers may be crossed by CuNPs, allowing for the targeted delivery of medications to the central nervous system in order to treat neurological illnesses [7]. Enhancing therapeutic agent accumulation through metal nanoparticle (NP) treatments, both by passive and active mechanisms, has been a significant focus of recent research. Passive targeting is largely based on the Enhanced Permeability and Retention (EPR) effect. Tumours and inflamed tissues exhibit leaky vasculature, allowing nanoparticles to accumulate. Recent studies highlight the effectiveness of this mechanism.

#### Enhanced permeability and retention (EPR) effect

The EPR effect facilitates the passive accumulation of nanoparticles in tumour tissues. A study by Maeda et al. [17] emphasises that the EPR effect remains a key factor in passive targeting, allowing metal NPs to preferentially accumulate in tumour tissues without external assistance.

#### NP size and surface characteristics

NPs in the range of 10–200 nm are shown to be ideal for leveraging the EPR effect, as larger particles can be excluded from normal tissues, while smaller particles are easily cleared by the kidneys [18].

Modifying NP surface charge and shape can further enhance their circulation time and accumulation.

Active targeting involves functionalizing NPs with ligands that bind to specific cell receptors.

#### Surface functionalization

In a recent study, it revealed metal NPs functionalized with targeting ligands such as folic acid and antibodies significantly improved binding to cancer cells expressing the corresponding receptors [19]. This increases the precision of drug delivery. Active targeting involves functionalizing nanoparticles with ligands (e.g. antibodies, peptides, or aptamers) that bind to specific receptors overexpressed on cancer cells, enhancing cellular uptake and improving specificity. A recent study shows gold nanorods conjugated with HER2 antibodies have shown improved targeting and therapeutic outcomes in breast cancer models [20]. These studies underline the dual approaches of passive and active targeting using metal NPs to improve therapeutic outcomes by enhancing drug accumulation at the desired site, while reducing off-target effects. All things considered, CuNP-based drug delivery systems show potential for enhancing the safety and effectiveness of therapeutic agents as well as resolving issues with traditional drug administration strategies.

### In biomedical imaging

In order to diagnose diseases, determine prognoses, and track their course of therapy, medical imaging is essential. Fluorescence imaging, photoacoustic imaging, computed tomography (CT), magnetic resonance imaging (MRI), and fluorescence imaging have all found use for copper nanoparticles as adaptable contrast agents [8]. CuNPs’ distinct optical and magnetic characteristics make it possible to utilise them as contrast agents with great sensitivity and resolution when visualising biological structures [4].

By altering the relaxation periods of the surrounding water molecules, CuNPs can act as T₁ or T₂ contrast agents in MRI, resulting in greater intensity of signal or signal restriction, respectively [4]. Similar to this, copper’s high X-ray attenuation coefficient makes it possible to see anatomical features with better contrast and spatial resolution in CT imaging [9]. Moreover, CuNPs may be functionalized with quantum dots or fluorescent dyes for fluorescence imaging applications, allowing for the very sensitive and selective real-time visualisation of biological processes [10].

Compared to conventional imaging agents, CuNP-based contrast agents have a number of benefits, such as biocompatibility, stability, and adjustable optical and magnetic characteristics [11]. Additionally, targeted imaging of sick tissues or molecular targets may be accomplished by conjugating targeting ligands to CuNPs, which enables early diagnosis and individualised therapy plans [12].

## Challenges and future perspectives

Even though green-synthesised copper nanoparticles for biomedical applications have advanced significantly, there are still a number of issues and restrictions that need to be resolved. These include problems with toxicity, biocompatibility, stability of nanoparticles, and regulatory approval. Furthermore, thorough preclinical examination and validation in pertinent animal models are necessary for the translation of laboratory-scale findings to clinical use [12].

The possible toxicity resulting from the emission of reactive oxygen species and copper ions is one of the main obstacles to the use of copper nanoparticles in biomedical applications. At low quantities, copper is a necessary micronutrient, but prolonged exposure to copper nanoparticles can cause cytotoxicity, oxidative stress, and inflammatory reactions [9].

A study reveals CuO NPs caused the mitochondrial membrane to depolarize and increased the formation of reactive oxygen species (ROS), which stopped the cell cycle in MDCK and AML-12 cells at the G2/M phase. This study was able to identify unique types of cellular death brought on by CuO NPs in these cell lines. AML-12 cells mostly showed early apoptosis, although MDCK cells combined autophagy and apoptosis [6]. Thus, to reduce any potential negative effects, meticulous synthesis parameter optimisation and comprehensive biocompatibility testing are crucial. The creation of scalable and repeatable synthesis techniques for environmentally friendly copper nanoparticle production is another difficulty. While several green synthesis methods have been documented in the literature, it is still very difficult to produce uniform nanoparticle shapes, sizes, and dispersibility [21]. To guarantee the repeatability and scalability of nanoparticle manufacturing, standardisation of synthesis techniques and optimisation of reaction conditions are crucial. Finding the ideal parameters for the synthesis of copper nanoparticles, such as pH, temperature, concentration ratio, and time, might be challenging for a researcher. The creation of nanoparticles is significantly influenced by time, while synthesis of copper nanoparticle A hue shift can happen in as little as 30 min in certain situations, or it can take up to 48 h. This uncertainty plays as major hindrance for green synthesis of copper [7].

Furthermore, thorough safety and effectiveness evaluations in compliance with international recommendations are required as part of the regulatory approval procedure for medicines and medical devices using copper nanoparticles. Enough preclinical research, such as toxicological and pharmacokinetic evaluations and clinical trials, are required to prove the efficacy and safety of products containing copper nanoparticles [22].

## Conclusion

The future of green-synthesised copper nanoparticles in biomedicine is bright, notwithstanding these obstacles. Copper nanoparticle-based solutions for disease detection, treatment, and prevention will continue to be innovatively designed and used thanks to advances in nanotechnology, materials science, and biomedical engineering. Furthermore, to expedite the translation of research results into clinical practice, multidisciplinary collaboration among investigators, practitioners, partners from the industry, and regulatory bodies is necessary. Through the delivery of nanoparticle for cancer detection, diagnosis, and therapy, nanotechnology has demonstrated a potential new era in cancer treatment. In the therapeutic context of several cancer types, cancer treatments based on the unique qualities of Nanoparticles are being widely employed. When compared to traditional medications, NP-based drug delivery system is associated with improved drug kinetics, biocompatibility, effect on tumour, and stability. Additionally, NPs offer a great platform for combination treatment, which aids in the recovery from drug resistance. Numerous nanoparticle forms, including metallic, hybrid, and polymeric NPs, have demonstrated increased drug delivery effectiveness as a result of increased study. More research should be directed into the nanoparticle research for its use into mass market treatment.

To sum up, green-synthesised copper nanoparticles have a great deal of promise to solve important biomedical issues such medication delivery, cancer treatment, antibiotic resistance, and medical imaging. Through the use of eco-friendly synthesis techniques and the distinctive physicochemical features of copper nanoparticles, scientists can create novel approaches to enhance patient welfare and improve healthcare results.

## Data Availability

All the hyperlinks of the reference data have been attached or quoted in the reference section.

## Abbreviations

CuNP: Copper nanoparticle
ROS: Reactive oxygen species
EPR: Enhanced permeability and retention
MDCK: The Madin-Darby canine kidney cell line
AML 12: Alpha mouse liver 12
